# Neuro-regeneration or Repair: Cell Therapy of Neurological Disorders as A Way Forward

**DOI:** 10.2174/1570159X22666240509092903

**Published:** 2024-06-27

**Authors:** Xiao-Yan Song, Cun-Xiu Fan, Atta-ur-Rahman FRS, Muhammad Iqbal Choudhary, Xiao-Ping Wang

**Affiliations:** 1 Department of Neurology, Shanghai General Hospital, Shanghai Jiao Tong University, School of Medicine, Shanghai, China;; 2 Department of Neurology, Jiading Branch of Shanghai General Hospital, Shanghai Jiao Tong University, School of Medicine, Shanghai, China;; 3 H.E.J. Research Institute of Chemistry, International Center for Chemical and Biological Sciences, University of Karachi, Karachi, Pakistan

**Keywords:** Cell therapy, stem cells, Parkinson’s disease, Alzheimer’s disease, amyotrophic lateral sclerosis, Wilson’s disease, stroke, traumatic brain injury

## Abstract

The human central nervous system (CNS) has a limited capacity for regeneration and repair, as many other organs do. Partly as a result, neurological diseases are the leading cause of medical burden globally. Most neurological disorders cannot be cured, and primary treatments focus on managing their symptoms and slowing down their progression. Cell therapy for neurological disorders offers several therapeutic potentials and provides hope for many patients. Here we provide a general overview of cell therapy in neurological disorders such as Parkinson’s disease (PD), Alzheimer’s disease (AD), amyotrophic lateral sclerosis (ALS), Wilson’s disease (WD), stroke and traumatic brain injury (TBI), involving many forms of stem cells, including embryonic stem cells and induced pluripotent stem cells. We also address the current concerns and perspectives for the future. Most studies for cell therapy in neurological diseases are in the pre-clinical stage, and there is still a great need for further research to translate neural replacement and regenerative therapies into clinical settings.

## 1 INTRODUCTION

The human central nervous system (CNS) has a limited capacity for regeneration and repair, as many other organs do. Accordingly, most neurological disorders cannot be cured, and primary treatments focus on managing their symptoms and slowing down their progression. The burden of disability and deaths caused by neurological disorders has progressively been recognized as a global public health challenge and is likely to become heavy owing to population growth and aging during the next few decades [1]. Therefore, there is a great need for urgent, effective treatment to reduce this burden.

Cell therapy for neurological disorders provides multiple therapeutic potentials *via* various mechanisms. First, stem cell therapy induces modifications that favor the restoration of endogenous cells since they generate factors or cytokines that modulate the response of the immune system, thus favoring endogenous repair. Second, the activation of endogenous cells may enhance the innate capacity of neurogenesis and angiogenesis, providing a reservoir of proliferating new cells by awakening the hibernating stem cells in the brain, promoting new cell growth, and accelerating stem cell migration to the damaged area [2]. Third, cell therapy can achieve systemic and local immunomodulation through an anti-inflammatory reaction, which may relieve the secondary cell death in the damaged tissue [3]. Fourth, cell transplantation achieves regeneration and repair by cell replacement or neural circuitry improvement. Exogenous cells contain stem cells and differentiated cells dedicated to a particular phenotype, including oligodendrocytes and astrocytes. Transplanted cells may integrate into their host network and provide subsequent circuit repair [4]. Cell therapy can also serve as a cell-based tool to develop new drugs and reveal the pathogenesis of various diseases.

**Fig. (1):**
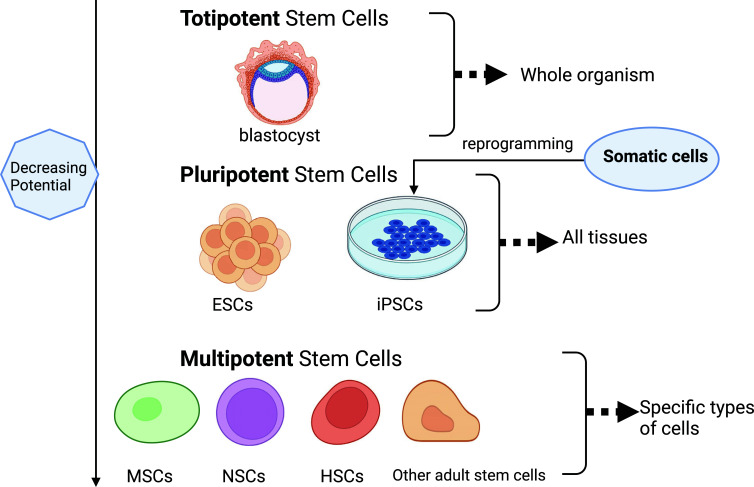
Stem cell classification: ESCs, embryonic stem cells; iPSCs, induced pluripotent stem cells; MSCs, mesenchymal stem cells; NSCs, neural stem cells.

Stem cells are characterized by the capacity to proliferate, self-renew, and differentiate into various cell lineages [5]. There are two types of human pluripotent stem cells: embryonic stem cells (ESCs) and induced pluripotent stem cells (iPSCs) (Fig. **1**). ESCs are derived from blastocysts and have a unique potential to develop into any type of human cell. iPSCs are adult cells that can be produced by treating somatic cells by reprogramming. iPSCs may be generated from a patient’s somatic cells (autologous) or those of another person (allogeneic), whereas ESCs are allogeneic. In addition, we can obtain stem cells of mesodermal, ectodermal, and mesodermal lineages from iPSCs, and NSCs, MSCs, or HSCs are also considered very promising stem cells.

In this review, we provide a general overview of cell therapy in neurological disorders such as Parkinson’s disease, Alzheimer’s disease (AD), amyotrophic lateral sclerosis (ALS), multiple sclerosis (MS), Wilson’s Disease(WD), stroke and traumatic brain injury (TBI). Then, the current concerns and perspectives for the future are discussed.

## 2 CELL THERAPY FOR NEUROLOGICAL DISORDERS

### 2.1 Parkinson’s Disease

Parkinson's disease (PD) is a progressive neurodegenerative disorder characterized by the loss of neurons in the nigrostriatal system. Patients with PD suffer from a combination of motor (*e.g*., tremor, rigidity, bradykinesia, and postural reflex disturbance) and non-motor symptoms (*e.g*., constipation, sleep disorder, and depression). Despite the established treatments, such as dopamine replacement therapy, along with other medications and surgical procedures, patients will inevitably develop disabilities and ultimately lose independence. In short, current treatments focus on controlling symptoms instead of improving the pathological condition [6]. Since PD pathology mainly lies in the loss of dopaminergic neurons, it is a good target for cell therapy.

Cell therapy for PD began in 1979 when Perlow and colleagues found that brain tissue grafts of dopaminergic neurons reduced motor abnormalities in the rat model of PD [7]. Thereafter, scientists have been pursuing the development of cell therapy for PD, both in experimental research and clinical study. Two clinical studies of fetal nigral cell transplantation in PD patients were published in 1988 [8, 9]. After that, these research studies were conducted. Freed and colleagues found that human embryonic dopamine-neuron transplants survive in 17 of 20 patients in the transplantation group, regardless of age and without immunosuppression. In a controlled trial, it was found that while this treatment resulted in some clinical benefits for younger patients, it did not show the same effectiveness in older patients [10]. The TRANSEURO trial was an open-label study in which patients with mild PD were randomly selected for transplantation of human fetal ventral mesencephalic tissue (hfVM) from a larger observational cohort [11, 12]. In these studies, positive findings were initially observed as the implants performed function as temporary trophic pumps. However, in some cases, later adverse events were observed, which either worsened the patient's quality of life or compromised his health. Due to the lack of hfVM and heterogeneity in grafts of hfVM tissues from several donors of different ages, surgery had to be canceled multiple times [13]. Besides, grafted NSCs can improve the content of Ceruloplasmin expression, which may play a neuroprotective role by decreasing iron deposition and ameliorating damage of dopaminergic neurons and possibly underline the iron-related common mechanism of Parkinson's disease [14].

For consideration of safer applications and medical ethics, scientists have been pursuing the development of other cell sources, such as ESCs, MSCs, NSCs, autologous dopaminergic cells, and other cells. Stem cells have the capacity of self-renewal and plasticity in the formation of a variety of tissues, which is needed for damaged nonrenewable neurons in PD. Thomson and colleagues first established human ESC lines from human blastocysts, which was a replacement for fetal tissue, and developed translational research toward clinical application [15]. Currently, several registered clinical trials are using human ESC-derived dopamine cells or human parthenogenetic NSCs as cell sources for transplantation [12, 16-19]. The concern with human ESCs for cell replacement still exists as with fetal tissues since these cells are also allogeneic and require immunosuppression to avoid graft rejection, while immunosuppression increases the risk of infections and side effects.

Then comes the era of the therapeutic strategy using autologous iPSC cell-derived neurons [20, 21]. The major merits of autologous cells are that they can avoid both the ethical issues associated with fetal and embryonic cells and immunological problems. Currently, several clinical trials have been conducted using human iPSC-derived cells in the treatment of PD [22, 23]. A patient with idiopathic Parkinson's disease was implanted with midbrain dopaminergic progenitor cells from the patient's source, and clinical measurements of postoperative Parkinson's disease symptoms stabilized or improved 18 to 24 months after implantation [18]. Professor Takahashi's team evaluated the safety and efficacy of dopaminergic progenitor cells (DAPs) derived from clinical-grade human iPSC lines and found that in rodent and monkey PD models, DA progenitor cells derived from human iPSCs could act as DA neurons without any side effects [19-21].

Cell therapies for PD have some limitations; for example, dopamine-related stem cell therapy can not improve nonmotor symptoms, and pathological changes can spread into grafted cells. The stem cells used, survival, differentiation, and integration into the host, reinnervation of the surrounding host milieu, the method of delivery and immunosuppression, and better trials all wait for further research [12]. Currently, 12 clinical trials of stem cell therapy for PD are underway (https://www.clinicaltrials.gov), including kinds of cell sources: ESCs, PSCs, MSCs, and NSCs, and there are no successful phase 3 trials yet.

### 2.2 Alzheimer’s Disease

Alzheimer’s disease (AD) represents another present-day social, economic, and medical crisis. Current drug therapy for AD is unable to halt the progression of neuronal degeneration [24]. The foundation laid by cell therapy for PD encouraged the researchers to study cell transplantation in AD. Stem cell transplantation into the brain can activate many therapeutic functions, including replacing the damaged tissue directly, secreting a host of neurotrophins to regulate neuroplasticity and neurogenesis, enabling antioxidant and anti-inflammatory activity, and modulating the immune system in the brain. It is worth emphasizing that positive events reported in preclinical studies are basically due to the anti-inflammatory modulation induced by their application, which may result in the expression of other agents, such as extracellular vesicles [25]. The anti-inflammatory modulation can induce a lower aggregation of amyloid oligomers and, consequently, greater connectivity and greater synapses.

Moghadam FH and colleagues found a substantial increase in cognitive function after engraftment with ESC-derived neuronal precursor cells (NPCs) and primed NPCs in an AD animal model [26]. Neuron-like cells derived from mouse ESCs transplantation improved AD cognition by improving neuronal connectivity [27]. Despite the ongoing research, there are similar issues with the current knowledge of ESCs, including ethical limitations, tumor formation, transplantation rejection, and immune response [28]. As mentioned before, iPSCs are becoming popular in degenerative diseases, including AD. Human-induced NPCs can reinforce hippocampal synaptic networks and rescue cognitive deficits in a mouse model of AD [29]. Comella-Bolla *et al*. demonstrated human pluripotent stem cell-derived NPCs could be functionally mature *in vitro* and integrated into the mouse striatum following transplantation [30]. NPCs integrated host environmental cues and differentiated them into striatal medium-sized spiny neurons, which successfully integrated into the endogenous circuitry without teratoma formation. NSC therapy, targeting both neuronal circuitry and pathological proteins to improve behavior and microenvironment, is also a promising treatment approach for AD [31]. Grafted NSCs into AD mice enhanced memory and learning by providing neurotrophic assistance [32]. Grafted modified NSCs helped replenish cholinergic neurons in the basal forebrain and shape hippocampal synapses and AchE fibers [33]. Interestingly, tracing of engrafted NSCs, including their survival, differentiation, and migration, is possible by 7.0 T of high-resolution MRI [34]. Apodaca *et al*. demonstrated that human NSC-derived extracellular vesicles could decrease dense core amyloid-β plaque accumulation in mice with AD [35].

Intravenous administration of MSCs could ameliorate cognition by promoting neurogenesis, decreasing oxidative stress, and upregulation of proteins related to neuronal synaptic plasticity [36]. Researchers also developed novel techniques to guide MSCs to the desired site of the brain [37, 38]. Jung *et al*. developed iron oxide nanoparticle-incorporated human Wharton’s jelly-derived MSCs to achieve a higher brain retention efficiency of MSCs under magnetic guidance [39]. In 2021, Kim *et al*. conducted a phase I clinical trial in nine patients with mild-to-moderate Alzheimer’s disease dementia by intracerebroventricular injections of human umbilical cord blood-derived MSCs. No serious adverse events occurred, and the symptoms of AD were mitigated [40]. Currently, several clinical trials of stem cell therapy for AD are ongoing (https://www.clinicaltrials.gov), with the majority of these trials focusing on MSCs. However, there are no successful phase 3 trials yet.

In sum, stem cell treatment has demonstrated exciting outcomes in AD studies even though risks still exist. More research and clinical trials, including cross-disciplinary research with other diseases, especially degenerative diseases, are still needed in the future.

### 2.3 Rare Disorders of Neurodegeneration: Amyotrophic Lateral Sclerosis, Wilson’s Disease and Multiple Sclerosis

#### 2.3.1 Amyotrophic Lateral Sclerosis

Amyotrophic lateral sclerosis (ALS) is another common progressive neurodegenerative disease characterized by muscle wasting, paralysis, and eventually death, which is mainly related to respiratory failure [41]. A limited number of drugs (riluzole and edaravone) approved for ALS cannot stop the progression of losing motor neurons (MNs). Just like PD, cell therapy is also an attractive treatment for ALS [42].

The two major promising therapeutic strategies for ALS are neuroprotection and neuronal replacement. Stem cells may perform immunomodulation, secrete growth factors, and produce supporting cells, including astrocytes, oligodendrocytes, and interneurons, which may protect damaged MNs from degeneration by providing a supportive environment [43]. Transplanted stem cells or stem-cell-derived neural progenitor cells can replace the damaged or dead MNs in the host and rebuild the motor control of voluntary muscles in ALS [44].

Various cell sources, such as NSCs, MSCs, ESCs, iPSCs, and hematopoietic stem cells (HSCs), have been used to treat ALS. Among them, iPSC-derived neural stem/progenitor cells are becoming popular because they may reduce the immune reaction and ethical problems related to the use of fetuses or embryonic tissues. Human iPSC-derived NSCs can survive and differentiate into neurons and glia after transplantation into the spinal cord, suggesting they can be a valuable alternative for autologous transplantation in clinical trials [45]. However, iPSCs carry an enormous risk of tumor formation. Accordingly, numerous studies have been conducted to improve iPSC reprogramming technology to reduce the risk of tumorigenesis and enhance the therapeutic applicability of iPSCs [46, 47]. In 2022, Lunetta *et al*. reported the result of a phase I/IIa clinical trial of autologous HSC transplantation in ALS, showing the transplantation was well tolerated, but it was not followed by any significant modification in disease progression [48].

The other concern about cell therapy for ALS is the delivery of stem cells. The location of the delivery of stem cells can affect the therapeutic effect. Suzuki *et al*. injected neural progenitor cells into four unilateral sites in the lumbar L1/L2 spinal cord in rats and found no MN-muscle contact and improvements in ipsilateral hind limb function [49]. When GDNF-secreting neural progenitor cells were injected into four unilateral sites at C3 and C6 spinal cord levels, protection to MNs and respiratory function were found [50, 51]. The motor cortex transplantation in rats was reported by Thomsen *et al*., showing delayed disease pathology, improved function, and increased life span [52]. Though it is feasible to transplant neural precursor cells into the brain elaborately using various new stereotactic devices, the best transplant route and location for ALS treatment still need to be studied further.

Khalid and Masroor reviewed clinical trials of stem cell treatment for ALS in children under 10, showing improvement in the overall survival chances. However, these early-phase trials did not show significant improvement in the rate of degeneration [53]. Gotkine and colleagues conducted a phase I/IIa clinical trial to evaluate the safety and therapeutic effects of intrathecal injection of AstroRx^®^ in patients with ALS [54]. AstroRx^®^ is an allogeneic cell-based product composed of healthy and functional human astrocytes derived from ESCs. The finding suggested that the administration of AstroRx^®^ was safe, with a signal of beneficial clinical effect observed for the first three months following injection.

#### 2.3.2 Wilson Disease

Wilson disease (WD) is an autosomal recessive disorder caused by genetic mutations in the ATP7B gene. WD is characterized by the pathological accumulation of copper, leading to a variety of clinical presentations, including liver failure, neurologic symptoms, and psychiatric manifestations. Pharmacological treatment for WD is a lifelong requirement, which can be challenging owing to drug side effects and patient adherence. Liver transplantation (LT) is another option for the treatment of WD. However, the shortage of liver donors and the requirement for lifelong immunosuppression limit its application. Besides, LT in patients with neurologic WD is still controversial. Fortunately, cell therapy raises hope for a permanent cure.

Hepatocyte transplantation is the most promising alternative to liver transplantation. Successful transplantation of healthy hepatocytes can integrate into liver parenchyma and restore deficient functions, including the transport of Cu into bile [55]. Several stem cells, especially MSCs, demonstrate the therapeutic potential for liver cell replacement [14, 56].

MSCs have promising potential for WD treatment. MSCs can differentiate into hepatocytes and can be used in allogeneic transplantation due to low immunogenicity. Besides, MSCs have the ability to move forward damaged areas under the signals released by the lesion, which makes it possible to achieve transplantation through many different ways, including intravenous, intraperitoneal, intrahepatic, or portal-venous injection [57]. MSCs can also secrete trophic factors to improve the restoration and regeneration of impaired liver [58]. Vanessa *et al*. found that ATP7B overexpression provided a selection advantage to MSCs in high copper microenvironments and might represent novel cell transplants for therapy of WD [59]. Then Zhang *et al*. reported that combination therapy with bone marrow mesenchymal stem cells (BMSCs) and penicillamine had a significant positive effect on liver fibrosis induced by hepatolenticular degeneration in a clinical trial [60]. Fujiyoshi *et al*. demonstrated that hepatocyte-like-cells, which were converted from stem cells out of human exfoliated deciduous teeth, achieved the function of copper excretion, thus offering a potential of functional restoring, bridging, and preventive approaches for treating fulminant WD [61]. Though considerable evidence suggests that MSCs infusion is attractive in treating WD, many issues need to be addressed, such as the shortage of donor organs, low cell engraftment, and a lack of long-lasting effects. It is essential to clarify the mechanism and related technology and, furthermore, to implement more verifications in preclinical trials.

In 2020, Wang *et al*. reported the generation of an induced iPSC line from a patient with WD harboring a homozygous Arg778Leu mutation in the ATP7B gene. This cell line had a normal karyotype, expressed pluripotency markers, and could differentiate into the three germ layers *in vivo* [62]. Another alternative therapy is the gene modification of ATP7B. Several pieces of literature have reported good outcomes in experimental animal models by using an infusion of recombinant adeno-associated virus-bearing ATP7B cDNA [63, 64]. Pöhler *et al*. reported that CRISPR/Cas9-mediated correction of ATP7B point mutations was feasible and might have the potential to be transferred to the clinic [65]. In 2022, Cai *et al*. established a system of autologous reprogrammed WD hepatocytes and achieved ATP7B gene therapy *in vitro* [66]. Then Liver progenitor cells-ATP7B-derived hepatocytes transplantation demonstrated therapeutic efficacy on copper homeostasis in a mouse model of WD.

With considerable studies about cell/gene therapy in WD, there is still a long way to achieve curative strategy in the clinical stage.

#### 2.3.3 Multiple Sclerosis

Multiple sclerosis (MS) is an autoimmune disease characterized by demyelination of white matter in the central nervous system. The currently available treatments are not recognized as curable options and mainly slow the progression of MS injuries to the CNS [67]. However, stem cell transplantation is emerging as a new option for treating MS. Currently, three distinct cell therapies are being carried out [68]. The first attempt is to use stem cells to replace oligodendrocytes formed by damaged myelin sheaths in the central nervous system. The second goal is to use hematopoietic stem cells to replace an individual's dysfunctional immune system. The third method attempts to utilize the endogenous stem cell population by mobilizing or not mobilizing *in vitro* amplification, utilizing its various repair and neuroprotective properties.

NPCs transplanted in animal models of MS have shown preclinical efficacy by promoting neuroprotection and remyelination by releasing molecules sustaining trophic support and neural plasticity. In experimental autoimmune encephalomyelitis (EAE), transplanted NPCs showed pathotropic properties migrating to demyelinating areas and inducing a rescue of the functional impairment in transplanted rodents. NPCs promote long-lasting neuroprotection through a bimodal mechanism: differentiating into mature brain cells with a reduction of demyelination, astrogliosis, and axonal loss and exerting trophic support and anti-inflammatory functions, maintaining undifferentiated features [69]. The prospective, therapeutic exploratory, non-randomized, open-label, single-dose-finding phase 1 clinical trial (NCT03269071, EudraCT 2016-002020-86), evaluating the feasibility, safety and tolerability of intrathecally transplanted human fetal NPCs (hfNPCs) in 12 patients with PMS in Italy [70]. Two groups of Italian researchers have presented data from their clinical trials using ESC in recent years [71]. Other groups have used MSC to modulate inflammation, and other groups have preclinically tested oligodendroglial lineage cells for demyelination. A variety of preclinical studies using the experimental autoimmune encephalomyelitis model of MS have recently shown that grafted cells with different origins, including MSCs, neural precursor and stem cells, and induced pluripotent stem cells, can repair CNS lesions and recover functional neurological deficits [72].

Hematopoietic stem cell transplantation (HSCT) represents a potentially useful approach to slow or prevent progressive disability in relapsing-remitting MS. Nonmyeloablative HSCT can result in prolonged time to disease progression. Further research is needed to replicate these findings and to assess long-term outcomes and safety [73]. Treatment with MSCs was well-tolerated in progressive multiple sclerosis and induced short-term beneficial effects regarding the primary endpoints, especially in patients with active disease [74].

### 2.4 Stroke

Stroke is the third leading cause of disability in adults worldwide. All the current treatments are not robust enough to completely restore function [75]. Initial research in cell therapy for stroke tried to replace neurons lost to vascular tissue injury. Fetal neocortical grafts implanted in brain infarcts in rats realized graft revascularization and ingrowth of afferent fibers from the host brain [76, 77]. Functional studies also demonstrated improved performance on behavior testing in rats by striatal grafts in striatum infarction [78]. Considering ethical concerns about the use of fetal tissue and resource scarcity, the research then transferred to more ethically acceptable sources, including human neuroteratocarcinoma (hNT) cells and fetal porcine cells. After the hopeful preclinical studies in rats, clinical trials turned out to be unsatisfactory because of the ineffectiveness and adverse events [79, 80]. The failure led the direction to target alternative mechanisms such as neurogenesis, angiogenesis, and immunomodulation. Studies of stem cell transplantation for stroke showed that functional improvements could occur without graft survival, indicating the therapeutic effect of stem cells attributed to the secretion of modulatory paracrine factors [81]. Stem cell lines have been used to exploit bystander effects by intravenous injection in the acute phase or intracerebral implantation in the chronic phase. Post hoc analysis of MASTETS demonstrated significantly excellent outcomes in the treatment arm [82].

Translational considerations have been discussed at Stem Cell Therapies as an Emerging Paradigm in Stroke (STEPS) meetings to establish consensus-based guidelines on the development of cell therapies for stroke [83, 84]. Two clinical trials, PISCES-II and A study of Modified Stem Cells in Stable Ischemic Stroke, tried to promote endogenous repair processes in the chronic phase by intracerebrally transplanting modified NSCs and marrow-derived MSCs, respectively, and the results turned out to be encouraging. Several clinical trials are still ongoing [85, 86]. Shichinohe and teammates reported to administrate autologous MSCs intracerebrally in the subacute phase of stroke and labeling grafted cells with superparamagnetic iron oxide for tracking the distribution of the transplanted cells over time [87].

In order to realize full function restoration, cell replacement is still being pursued. The first challenge is the poor survival of grafted cells. The preclinical research sought to enhance survival by modifying the cells, such as hypoxic preconditioning and genetic modification, or protecting them from the post-transplant environment [88-90]. The second challenge is how to recapitulate the complicated architecture of brain tissue and the precise neural circuitry in the damaged brain. Thus, electrophysiological studies are needed to elaborate on the specific mechanisms. There is more work to be done across the field.

Moniche *et al*. reported the result of phase 2, a randomized, open-label, multicentre trial about the safety and efficacy of intra-arterial bone marrow mononuclear cells (BMMNCs) transplantation in patients with acute ischaemic stroke in Spain (IBIS trial). The finding showed that intra-arterial BMMNCs were safe, but there was no significant improvement at 180 days on the mRS [91]. It seems that cell therapy for stroke is needed to ameliorate systemic and local inflammation in the acute phase and to achieve cell engraftment in the chronic phase. Both pre-clinical and clinical research are still expected in the near future.

### 2.5 Traumatic Brain Injury

Traumatic brain injury (TBI) is a leading cause of mortality and morbidity [92]. TBI is caused by physical trauma to the brain and can lead to long-lasting dysfunctions. Common events that can cause TBI include falls, vehicle-related collisions, violence, sports injuries, explosive blasts, and other combat injuries. There is no effective therapy for TBI except for rehabilitation and some palliative drugs. TBI was previously recognized as an acute injury due to the lack of understanding of the chronic functional deficits, but it is now considered a chronic disease because of the secondary dysfunction that accompanies the initial insult [93]. Long-term inflammatory cascade initiated by TBI may persist and expand in the brain, predisposing TBI patients to neurodegenerative diseases and mental health disorders [94, 95]. Since they can provide a neuroprotection role and help reconstruct damaged tissues, stem cells are used as a potential therapeutic option for the treatment of TBI [96]. Several cell types have been studied for TBI therapy, such as adipose tissue-derived MSCs, bone marrow-derived MSCs, and umbilical cord-derived MSCs [97].

Initially, stem cell transplants were thought to function in the CNS by completely replacing damaged neural cells with new cells. However, the planted stem cells survived poorly in the damaged tissue of the host, while functional recovery was observed [98]. Therefore, there must be more complicated mechanisms under stem cell therapy. As mentioned above, in other CNS diseases, transplanted stem cells may secrete neurotrophic factors play a part. These neurotrophic factors tend to achieve therapeutic effects by activating cell survival pathways, yet their expression generally decreases in TBI [99]. Studies about administering stand-alone neurotrophic factors revealed various complications, and its clinical application is limited [100, 101]. By contrast, stem cells are capable of responding to the real-time environment and secreting appropriate neurotrophic factors. In 2021, Kawabori *et al*. demonstrated chronic motor deficits secondary to TBI could be improved significantly by implantation of allogeneic-modified bone marrow-derived MSCs [102].

Stem cell transplants also may play an indirect role in activating and amplifying the natural neuroprotective responses that may otherwise remain latent. Research has found that some endogenous stem cells, although with restrained capacity, may promote neurogenesis under the right conditions. This discovery suggests potential therapeutic strategies for brain injury [103, 104]. Beyond the hippocampal dentate gyrus and subventricular zone of the lateral ventricles, more identified sites of adult neurogenesis, such as the meninges and circumventricular organs, are also possible targets for endogenous repair [105]. Exogenous stem cell transplants could facilitate the lengthy migration of endogenous stem cells. Tajiri N and colleagues revealed that long-distance migration of host cells from the neurogenic niche to the injured brain site could be achieved through transplanted stem cells serving as bio bridges for the initiation of endogenous repair mechanisms [106]. The study indicated that transplanted stem cells and endogenous stem cells could band together to improve TBI damage.

The main source of exosomes is the secretome of stem cells, which can transport and deliver a large cargo of proteins, lipids, and nucleic acids and can modify cell and organ function [107]. Moreover, the secretome of stem cells appears to be of greater benefit compared to the cells themselves. The transplanted exosomes are another possible mechanism by which stem cell transplants apply their indirect therapeutic effects after TBI. Human MSC-generated exosomes significantly improve functional recovery in rats after TBI, at least in part, by promoting endogenous angiogenesis and neurogenesis and reducing neuroinflammation [108]. Furthermore, MSCs could secrete bioactive factors to stimulate neurogenesis and improve outcomes of TBI in a rat model [109]. MSC secretome alone has been found to enhance endogenous neurogenesis [110]. Exogenous NSC transplantation was also linked to the reorganization of endogenous neural progenitor process projection [111].

The clinical studies about cell therapy after TBI were mostly small and uncontrolled, but the results showed positive effects [112]. In 2020, Sharma *et al*. demonstrated the safety and efficacy of autologous BMMNCs transplantation in 50 patients with chronic TBI on long-term follow-up [113]. Another important clinical trial was the 1-year, randomized, double-blind, controlled, Phase-2 STEMTRA study [102]. Sixty-one patients were treated with allogeneic-modified bone marrow-derived mesenchymal stromal cells or sham control, and the primary efficacy endpoint of significant improvement was achieved at six months. There were no deaths or withdrawals due to adverse events. Further research will focus on optimizing cell sources, different cell doses, method of delivery, and specific patient characteristics such as age and the phase of TBI.

## 3 CHALLENGES AND PROSPECTIVES

A growing number of cell therapies demonstrate great potential to be revolutionary treatments for many CNS diseases. However, several challenges must be addressed. First and foremost, many studies cited use rodent models for evaluation of cell engraftment, and extant models cannot mimic the pathogenesis of human CNS diseases accurately. The major problem is to clarify how stem cells work in the host body and how they integrate with the targeted tissue network successfully. The generated specialized cell typologies should adapt to the host environment.

The Safety of cell therapy should be considered, including concerns such as genetic instability after long-term expansion and stem cell migration to other regions or organs. Autologous patient-derived cells may circumvent ethical concerns, but genetic engineering or reprogramming these adult cells to amplify stemness can lead to uncontrolled proliferation and genetic abnormality. Allogeneic healthy cells may avoid the disease phenotype of cell source but face the risk of immune-mediated rejection. Developing validated *in vitro* and *vivo* model systems is essential to improve the longevity and differentiation potential of stem cells in CNS. As for clinical trials, there are other safety considerations, such as the potential for malignant transformation and side effects, including epilepsy and immune allergic reactions, should be noted.

Clarifying cost-effective and appropriate conditions to culture those cells is also not an easy task. Besides that, optimized cell dose, the best route of administration, and the target site are also crucial to better outcomes. Since most of the data were derived from animal studies, administering the strategy to a heterogeneous patient population should be prudent.

Currently, it seems unrealistic to replace lost neurons with grafted cells and integrate them into existing neural circuitry. However, delivering therapeutic factors and delaying the disease progression by grafted cells may be a short-term achievable goal. From the laboratory to the clinical field, there is still a long way to go. The practice regulations for stem cell therapies are being used to supervise clinical trials.

## CONCLUSION

All the CNS disorders mentioned above cannot be cured with conventional treatments, and the probability of stopping those diseases’ progression and regenerating damaged neurons makes cell therapy promising and exciting. Tailoring different types of stem cells to repair the specific defect in each neurological disorder is essential. More pre-clinical research and clinical trials with standard protocols are pursuing to translate neural replacement and regenerative therapies into clinical settings.

## LIST OF ABBREVIATIONS

AD: Alzheimer’s Disease
ALS: Amyotrophic Lateral Sclerosis
BMMNCs: Bone Marrow Mononuclear Cells
CNS: Central Nervous System
ESCs: Embryonic Stem Cells
HfVM: Human Fetal Ventral Mesencephalic Tissue
HLA: Human Leukocyte Antigens
HSCs: Hematopoietic Stem Cells
iPSCs: induced Pluripotent Stem Cells
LT: Liver Transplantation
MNs: Motor Neurons
MS: Multiple Sclerosis
MSCs: Mesenchymal Stem Cells
NPCs: Neuronal Precursor Cells
NSCs: Neural Stem Cells
PD: Parkinson’s Disease
TBI: Traumatic Brain Injury
WD: Wilson’s Disease
